# Pushing the Surgical Limits: Primary Total Knee Arthroplasty Using Rotational Prosthesis in a 96-Year-Old Lady with End-Stage Osteoarthritis

**DOI:** 10.7759/cureus.13294

**Published:** 2021-02-11

**Authors:** Natasha G, George Hourston, Azeem Thahir, Andrew Carrothers

**Affiliations:** 1 Trauma and Orthopaedic Surgery, Addenbrooke’s Hospital, Cambridge University Hospitals NHS Foundation Trust, Cambrige, GBR; 2 Trauma and Orthopaedic Surgery, Addenbrooke’s Hospital, Cambridge University Hospitals NHS Foundation Trust, Cambridge, GBR

**Keywords:** arthroplasty, severe osteoarthritis, nonagenarian population

## Abstract

We present the case of a 96-year-old patient treated for severe osteoarthritis with primary total knee arthroplasty (TKA) using a rotational prosthesis. The patient had significant medical comorbidities and her independence was limited due to her severe functional immobility. This case demonstrates that TKA can be a safe procedure with good outcomes in nonagenarians with severe osteoarthritis. Thorough discussion of treatment options is crucial for elderly patients with multiple medical comorbidities. TKA in the nonagenarian population can restore function and independence for patients which may reduce the burden on social care.

## Introduction

Owing to a rise in life expectancy, there is an unprecedented increase in the number of octo- and nonagenarians (people aged between 80 and 99 years) with advanced osteoarthritis. Intuitively, orthopedic surgeons regard the risk for complications in this age group to be greater. Due to poorer physiological reserve, delayed wound healing, and preexisting multiple medical comorbidities, elderly patients often face delays in preoperative optimization and are also at higher risk of postoperative complications. Yet, with improved surgical technology, general anesthesia, and geriatric care, the proportion of patients with successful, uncomplicated total knee arthroplasty (TKA) is on the rise, which markedly improves quality of life.

## Case presentation

A 96-year-old lady was referred to the orthopedics outpatient clinic with worsening left knee pain secondary to advanced osteoarthritis. Her pain, which occurred even at rest, was constant despite optimized pain medications and significantly affected her independence and quality of life. She mobilized at home with a walking stick, but her mobility was limited by pain and deformity. She denied any hip pain. Her past medical history included critical aortic stenosis, dual-chamber permanent pacemaker inserted for trifascular block in 2012, previous bilateral total hip and shoulder replacements, and a right TKA. She lived alone, mobilized with a frame, and was independent for all activities of daily living.

On examination, she had an antalgic gait with varus thrust. The fixed varus deformity of her left knee was approximately 45° clinically, with a fixed internal rotation deformity of her tibia. She also had a fixed flexion deformity of 25° and a contracted medial collateral ligament and loose lateral collateral ligament. Her extensor mechanism was intact with a range of movement from 20° to 110°. Her peripheral pulses were palpable and sensation was intact.

**Figure 1:**
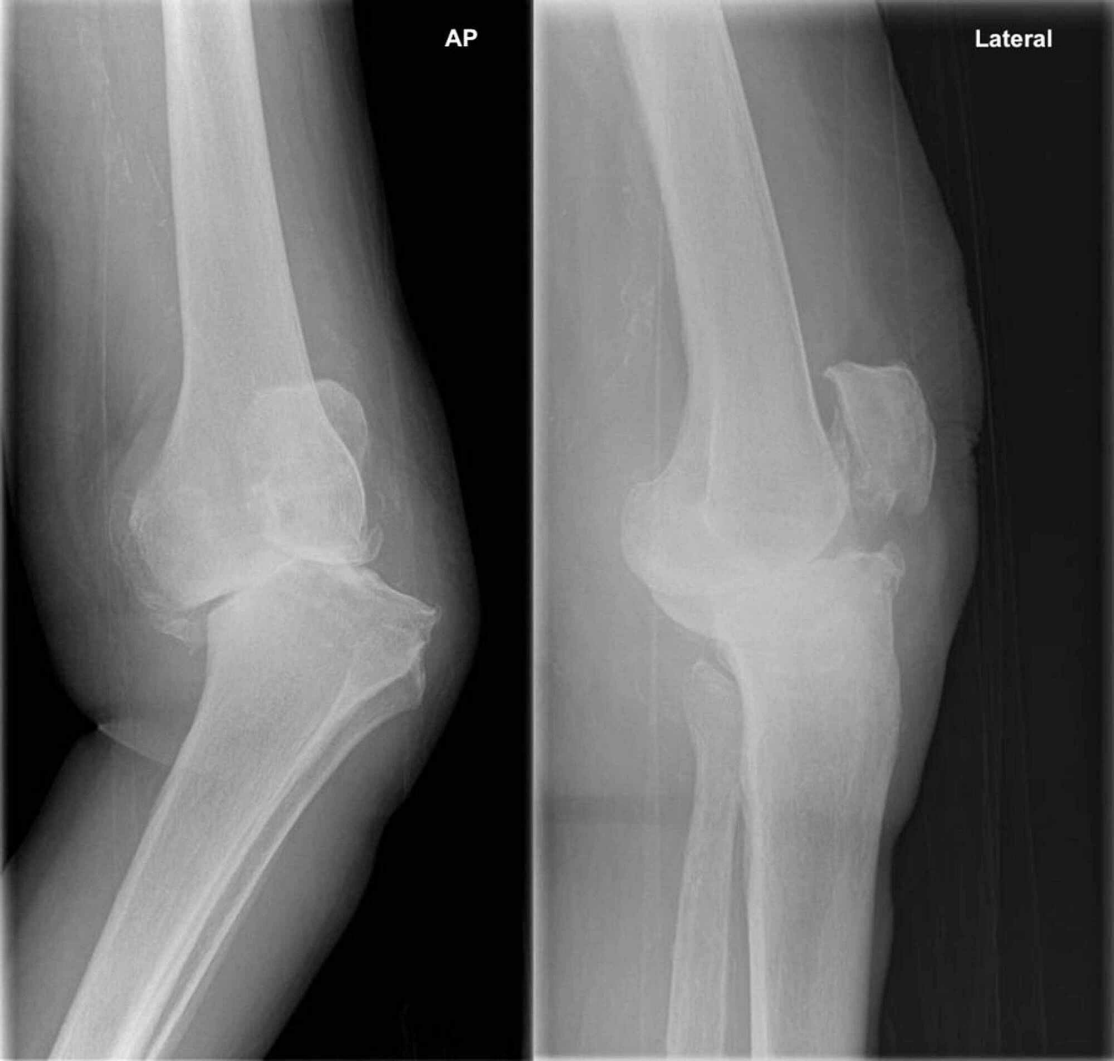
Anterior-posterior and lateral radiographs of the left knee revealing end-stage osteoarthritis with varus deformity.

Plain anterior-posterior and lateral X-rays of her left knee revealed features of tricompartmental osteoarthritis, including loss of joint space, osteophytes, subchondral cysts, subarticular sclerosis, and significant varus deformity (Figure 1). There was significant bone loss, mostly on the medial tibial plateau, with subluxation of the knee joint. Plain X-rays of her left hip revealed that bone stock in the femur was well preserved.

**Figure 2:**
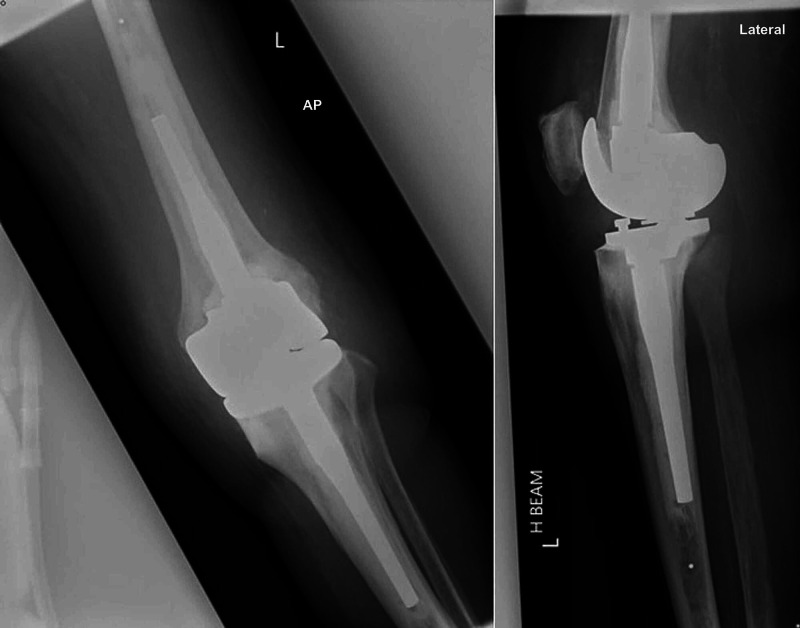
Anterior-posterior and lateral radiographs of the left knee post-TKA using a rotating hinge endolink. TKA, total knee arthroplasty

Given her excellent functional baseline but bearing in mind her age, both non-operative and operative management approaches were discussed with the patient. The benefits and risks of a TKA were discussed, including infection (potentially necessitating second stage revision), venous thromboembolism, revision surgery, nerve or vessel damage, fractures, anesthetic complications, and death. The patient opted for operative management and we proceeded with a complex total left knee arthroplasty using a rotating hinge endolink due to her significant varus deformity under general anesthesia (Figure 2). Her preoperative echocardiography revealed critical aortic stenosis with preserved left ventricular function. This was treated with a transcatheter aortic valve implantation without complications, and she was treated medically with apixaban. Her apixaban was stopped preoperatively, and her pacemaker check was normal.

Intraoperatively, care was taken to use only short bursts of mono-polar diathermy for hemostasis due to her indwelling pacemaker.

She recovered without complications postoperatively, had satisfactory blood examination and X-rays, and underwent physiotherapy. She restarted her apixaban without bleeding complications. Notably, she was hyponatremic with a sodium level of 124 mEq/L. On assessment, she was hypovolemic. Hyponatremia screen revealed normal early morning cortisol and thyroid function tests. Both serum osmolality and urinary sodium were low at 265 mmol/kg and <10 mEq/L, respectively. This was discussed with the endocrinology team who agreed that the hyponatremia was likely secondary to dehydration postoperatively. Her sodium improved to 131 mEq/L with intravenous fluids.

The patient was discharged from the ward after a five-day stay, extended only due to her electrolyte abnormality, and received community physiotherapy. She has subsequently been followed up in clinic with good recovery and her Oxford Knee Score six months postoperatively was 54.

## Discussion

The nonagenarian population is increasing with improved health and social care. Degenerative conditions such as osteoarthritis are, therefore, becoming increasingly prevalent. A recent study investigating TKA for nonagenarians in Sweden found good outcomes for death and revision rates, as well as patient-reported outcomes compared with other age groups undergoing TKA [1]. Complications after TKA have been reported at between five out of 20 and 11 out of 15 TKAs [2,3]. Reported postoperative complications among this age group include major complications such as myocardial infarction and stroke, as well as less serious complications such as confusion and pressure sores. Importantly, post-TKA complication rates among the nonagenarian population seem to be higher than younger patients, although these are frequently medical complications that resolve in the early postoperative period. Given their prompt resolution, most such complications do not impact the general quality of life and independence of these patients in the short-to-medium term. Longer-term follow-up including mortality rate is less easily interpreted among this elderly group because these patients have a short life expectancy. Some authors suggest that death rates up to and including one year postoperatively are better indicators of safety among this age group [1].

The use of implants with a rotational hinge component in TKA is commonly reserved for complex revision procedures. Indications for rotating hinge implant in primary TKA include collateral ligament insufficiency, severe valgus/varus deformity (greater than 20°) with relevant soft-tissue release, relevant bone loss, including collateral ligament insertions, gap imbalance in flexion-extension, ankylosis, and hyperlaxity [4]. In this unusual case, such a device was implemented during the primary procedure. It has been noted in the literature that this is an appropriate salvage device in appropriate patients with good outcomes at the mid-term follow-up [5].

Cost-benefit considerations must also be made when undertaking elective surgery for the elderly population. Length of hospital stay for acute postoperative care has been reported as higher among the nonagenarian population at around 10-15 days, with associated increase in the cost of TKA [1]. Offsetting this amount with the public health cost of keeping a healthy and active patient population from needing TKA could be deliberated. One group compared the costs of elective TKA with the costs of nursing home placement and found favorable results when opting for surgery [6]. Clearly, this is an important consideration in the context of a nationally funded health and social care service when arthritis and the associated immobility contribute to 15% of all nursing home placements among residents without dementia [7].

## Conclusions

TKA is a safe procedure with good outcomes in nonagenarians with severe osteoarthritis. Thorough discussion of treatment options is crucial even for elderly patients with multiple medical comorbidities. TKA in the nonagenarian population can restore function and independence for patients which may reduce the burden on social care.
